# Co-Loading of Temozolomide and Curcumin into a Calix[4]arene-Based Nanocontainer for Potential Combined Chemotherapy: Binding Features, Enhanced Drug Solubility and Stability in Aqueous Medium

**DOI:** 10.3390/nano11112930

**Published:** 2021-11-02

**Authors:** Rossella Migliore, Nicola D’Antona, Carmelo Sgarlata, Grazia M. L. Consoli

**Affiliations:** 1 Istituto di Chimica Biomolecolare, Consiglio Nazionale delle Ricerche, Via Paolo Gaifami 18, 95126 Catania, Italy; rossella.migliore@unict.it (R.M.); nicola.dantona@icb.cnr.it (N.D.); 2 Dipartimento di Scienze Chimiche, Università degli Studi di Catania, Viale Andrea Doria 6, 95125 Catania, Italy

**Keywords:** calix[4]arene-based nanoparticle, temozolomide, curcumin, drug delivery, binding interactions, drug combination

## Abstract

The co-delivery of anticancer drugs into tumor cells by a nanocarrier may provide a new paradigm in chemotherapy. Temozolomide and curcumin are anticancer drugs with a synergistic effect in the treatment of multiform glioblastoma. In this study, the entrapment and co-entrapment of temozolomide and curcumin in a *p*-sulfonato-calix[4]arene nanoparticle was investigated by NMR spectroscopy, UV-vis spectrophotometry, isothermal titration calorimetry, and dynamic light scattering. Critical micellar concentration, nanoparticle size, zeta potential, drug loading percentage, and thermodynamic parameters were all consistent with a drug delivery system. Our data showed that temozolomide is hosted in the cavity of the calix[4]arene building blocks while curcumin is entrapped within the nanoparticle. Isothermal titration calorimetry evidenced that drug complexation and entrapment are entropy driven processes. The loading in the calixarene-based nanocontainer enhanced the solubility and half-life of both drugs, whose medicinal efficacy is affected by low solubility and rapid degradation. The calixarene-based nanocontainer appears to be a promising new candidate for nanocarrier-based drug combination therapy for glioblastoma.

## 1. Introduction

The co-vehiculation of drugs by a nanocarrier is currently being investigated as a strategy that might expand successful method that can be used to battle tumors [1]. By modulating different signaling pathways in cancer cells and causing synergetic responses or additive effects, the combination of two or more drugs can maximize the therapeutic efficacy and overcome tumor drug resistance phenomena [2]. Compared to the direct co-administration of free drugs, the co-vehiculation by a nanocarrier provides the advantage of unifying the pharmacokinetics of each drug as well as the benefits of a nanocarrier-based drug delivery, including protection from premature degradation, an increase of solubility and bioavailability, prolonged circulation time, the ability to cross biological barriers, delivery to the targeted disease site, modulation of drug release profile, and the minimization of adverse effects [3]. 

Among the variety of drug mixtures, the combination of temozolomide (TMZ) and curcumin (CUR) has been investigated as a more effective treatment of multiform glioblastoma (GBM), which is the most common and aggressive malignant primary brain tumor that involves various molecular pathways. It has been reported that the co-administration of CUR and TMZ improves therapeutic response to TMZ. CUR sensitizes glioblastoma to TMZ treatment [4] and promotes TMZ-induced apoptosis [5] whilst the CUR/TMZ combination exhibits more cytotoxicity than the individual drugs do in glioblastoma cells [6]. The loading of TMZ and CUR in a suitably structured nanocarrier is advantageous for combined therapy and is essential for overcoming limitations such as rapid TMZ degradation [7] and CUR low water solubility and ease degradability [8] in physiological conditions.

**Figure 1:**
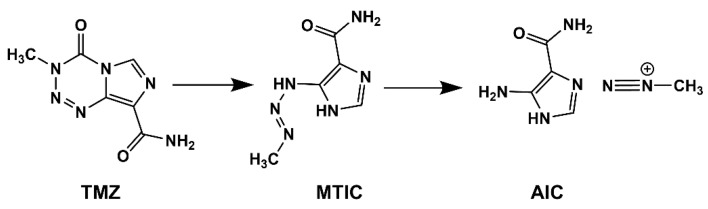
TMZ degradation pathway.

TMZ, the first-line chemotherapeutic agent for GBM, is a prodrug for 5-(3-methyltriazen-1-yl)imidazole-4-carboxamide (MTIC), the active alkylating agent that damages DNA through the transfer of a methyl group to the N-7 or O-6 position of the guanine residues leading to inhibition of DNA replication and cell-cycle arrest [9,10,11]. Under slightly alkaline conditions, TMZ is rapidly hydrolyzed to MTIC (Figure 1), which rapidly degrades to the methyl diazonium cation and 5-aminoimidazole-4-carboxamide [12]. 

As TMZ has a short half-life of 1.8 h [13] and since MTIC has poor blood–brain barrier penetration and reduced cellular uptake, high doses of TMZ need to be repeatedly administered to achieve the desirable antitumor effect. However, prolonged therapy leads to TMZ resistance and poor responsiveness to subsequent treatments. The entrapment of TMZ in the cavity of macrocycles, such as cyclodextrin [14,15], cucurbituril [16], or *p*-sulfonato-calix[4]arene [17], and in nanostructured systems, including liposome [18], lactoferrin nanoparticle [19], and lipidic nanocarriers [20], has enhanced the chemotherapeutic efficacy of TMZ by increasing its half-life and consequently the amount of drug gaining in the target cells. 

CUR is a natural ingredient with multiple medicinal effects [21]. Preclinical in vitro and in vivo data have shown that CUR elicits its own anticancer activity on GBM (induction of G2/M cell cycle arrest, activation of apoptotic pathways, induction of autophagy, disruption of molecular signaling, inhibition of invasion, and metastasis) [22] and enhances the efficacy of other drugs [23], including TMZ. The entrapment in a variety of nanocarriers [24] has been explored for overcoming the low solubility, stability, and bioavailability of CUR that compromise its therapeutic efficacy.

**Figure 2:**
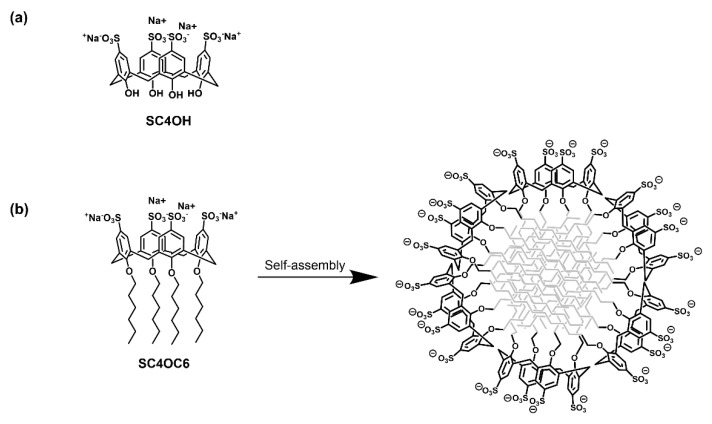
(**a**) Structure of the *p*-sulfonato-calix[4]arene-OH (SC4OH); (**b**) structure of the *p*-sulfonato-calix[4]arene-*O*-hexyl (SC4OC6); and schematic representation of its self-assembly in a micellar nanoaggregate.

Calix[n]arenes are a family of macrocyclic oligomers that are formed by phenolic units linked by methylene bridges and have gained interest as drug delivery systems [25,26]. Calix[n]arene derivatives can enhance the water solubility, stability, and efficacy of a variety of drugs through complexation in their hydrophobic cavity and/or entrapment in nanoaggregates obtained from the self-assembling of amphiphilic calix[n]arenes that have been properly functionalized with a variety of hydrophilic and hydrophobic moieties [25,26,27]. *p*-Sulfonato-calixarenes are promising candidates for biomedical applications due to their high water-solubility, low toxicity and immunogenicity, and biocompatibility [28,29]. In a monomeric or nanoassembled form, they have been proposed as efficient carriers for a variety of drugs [28,29,30,31]. Recently, it has been reported that the inclusion of TMZ into the cavity of a monomeric *p*-sulfonato-calix[4]arene-OH (SC4OH, Figure 2a) protected the drug from the rapid hydrolysis and enhanced its efficacy against glioblastoma in in vitro and in vivo experiments [17]. In the search for novel nanocarriers to be potentially employed for a drug-combined therapy against multiform glioblastoma, we investigated the capability of an amphiphilic *p*-sulfonato-calix[4]arene-*O*-hexyl ether (SC4OC6, Figure 2b) with the ability to form micellar nanoparticles to load TMZ and CUR both singularly and in combination in aqueous medium at neutral pH. UV-Vis and Nuclear Magnetic Resonance (NMR) spectroscopy, Isothermal Titration Calorimetry (ITC), and Dynamic Light Scattering (DLS) were used for the characterization of the binary (SC4OC6/TMZ and SC4OC6/CUR) and the ternary (SC4OC6/CUR/TMZ) nanostructured systems, thus highlighting the improved features (such as solubility and half-life) of the encapsulated drugs.

## 2. Materials and Methods

### 2.1. Reagents

All chemicals and solvents were purchased from Sigma-Aldrich (Milan, Italy) and were used without purification. The tetra-hexyloxy-*p*-sulfonato-calix[4]arene (SC4OC6) was synthesized using the procedure reported in the literature [32]. Curcumin (CUR), temozolomide (TMZ), and *p*-sulfonato-calix[4]arene (SC4OH) were purchased from Sigma-Aldrich and were used without purification. Buffer solutions (10 mM) were prepared by weighing the proper phosphate salts (Sigma-Aldrich), and their pH value was controlled potentiometrically. High purity water (Millipore, Milli-Q Element A 10 ultrapure water) and A grade glassware were employed throughout.

### 2.2. Instrumentation

^1^H NMR (400.13 MHz) spectra were acquired on a Bruker Avance 400 spectrometer (Bruker Scientific LLC., Billerica, MA, USA). Chemical shifts (*δ*) are expressed in parts per million (ppm) and are reported relative to the residual water proton peak. Lyophilization was performed on Lyoquest-85 instrument, Telstar (Telstar, Terrassa, Spain). UV–vis spectra were recorded on an Agilent Technologies 8453 UV−vis spectrophotometer (Agilent Technologies, Santa Clara, CA, USA), and fluorescence spectra were acquired on a Horiba-Jobin-Yvon Fluoromax-3 fluorescence spectrometer (Horiba Instruments Inc., Piscataway, NJ, USA). Size and zeta potential measurements were performed on a ZetaSizer NanoZS90 Malvern Instrument (Malvern Panalytical Ltd., Malvern, UK) equipped with a 633 nm laser at a scattering angle of 90° and at 25 °C. The calorimetric measurements were run on an actively controlled (power compensated) nano-ITC calorimeter from TA Instruments (New Castle, DE, USA). 

### 2.3. Synthesis and Characterization of SC4OC6

To a solution of SC4OH (1.0 g, 1.3 mmol) in water (5 mL) and DMSO (20 mL), NaOH (1.0 g, 25 mmol) and 1-bromohexane (4 mL, 29 mmol) were added. The mixture was stirred at 50 °C for 24 h. After cooling, MeOH was added to obtain a precipitate that was collected by filtration. The solid was dissolved in water (5 mL) and was precipitated again by the addition of EtOH (three times) [32]. ^1^H-NMR (400 MHz, DMSO-*d6*): δ 0.89 (t, 12 H, *J* = 6.4 Hz, 4 × CH_3_), 1.35 (br s, 24 H, 12 × CH_2_), 1.92 (s, 8 H, 4 × CH_2_), 3.23 and 4.33 (AX system, 8 H, *J* = 12.6 Hz, 4 × ArCH_2_Ar), 3.87 (t, 8 H, 4 × OCH_2_), and 7.18 (s, 8 H, 4 × ArH).

### 2.4. Preparation and Characterization of the Micellar SC4OC6 Nanoparticles

The self-assembly of SC4OC6 in micellar nanosized aggregates occurred by the simple dissolution of the compound in a phosphate-buffered solution. The size of the micellar nanoparticles was detected by DLS at 25 °C, and the critical micellar concentration (CMC) was determined by ITC measurements.

### 2.5. ITC Titrations

ITC measurements were run at 25 °C using a nano-isothermal titration calorimeter with an active cell volume of 0.988 mL and a 250 μL injection syringe. During the titration, a stirring rate of 250 rpm was employed. Measurements were conducted in the overfilled mode, which overcame issues related to liquid evaporation and the presence of the vapor phase [33]. NanoAnalyze software ((NanoAnalyze Data Analysis Version 3.12.0, TA Instruments, New Castle, DE, USA)) was used to integrate the power curve and to obtain the gross heat evolved/absorbed in the reaction. The calorimeter was calibrated chemically through the procedure previously described [34]. An electrical calibration was also conducted. 

*Micelle formation:* A total of 2–3 independent (de-)micellization experiments [35] were run to obtain accurate values for both the critical micellar concentration (CMC) and the enthalpy of micellization (ΔH_mic_) for the amphiphilic calixarene. In these experiments, a SC4OC6 solution (1 mM) was titrated into the reaction cell containing buffer solution only. The concentration of the calixarene in the syringe (titrant) was chosen in such a way that the surfactant concentration in the measurement cell progressively increased and reached the CMC value during the experiment; the analysis of the heat values recorded allowed for the direct determination of the CMC and ΔH_mic_ parameters.

*Drug-micelles interaction:* The ITC measurements for the study of the interactions of TMZ with the micellar aggregates were conducted by titrating a solution of TMZ (4–6 mM) into a SC4OC6 solution (0.2–0.3 mM) well above its critical micellar concentration, e.g., at a concentration in which SC4OC6 is in the aggregated form. The same conditions were used for the titrations of TMZ into either SC4OC6 nanoaggregate loading curcumin or the non-amphiphilic SC4OH receptor. ITC experiments were also conducted by titrating a solution of TMZ (5 mM) into a solution of SC4OC6 in its monomeric form (20 μM).

Properly weighed amounts of the compounds were dissolved in 10 mM phosphate buffer to reproduce neutral conditions and to minimize any heat contribution resulting from the interaction of the compounds with the proton. Three independent titrations were run for each system to collect a proper number of points for a satisfactory fit of the curves. Blank experiments were conducted by titrating TMZ solutions (prepared in phosphate buffer) into a solution containing phosphate buffer only to determine the dilution heat.

Subtracting the heat evolved/absorbed in the blank experiment from the gross heat allowed us to obtain the net heat of the reaction, which was analyzed by HypCal [36]. This software enabled the determination of the stability constants and enthalpies for the formation of host–guest or micelle–guest adducts in solution by a non-linear least-squares minimization of the function

$$U=\sum {\left(Q_{obs.\;}-Q_{calc.}\right)}^{2}$$

where *Q_obs._* is the observed heat for a given reaction step and corrected for the blank, while *Q_calc_* is calculated as

$$Q_{calc.}=-\sum \delta n∆H$$

where *δn* is the change in the number of moles of a reaction product and Δ*H* is the molar formation enthalpy of the reaction product. The sum included all of the reaction steps. The squared residuals ${\left(Q_{obs.\;}-Q_{calc.}\right)}^{2}$ are summed over all of the titration points. The simultaneous analysis of the calorimetric data obtained from different titrations enabled us to obtain the binding parameters. 

### 2.6. Loading of TMZ in Micellar SC4OC6 Nanoparticles

A solution of SC4OC6 (36.3 mg, 31 mM) in 10 mL phosphate buffer (10 mM, neutral pH) was added to solid TMZ (6.1 mg). The suspension was vortexed for 5 min and was shaken at 300 rpm for 1 h at room temperature and was then centrifuged at 6000 rpm for 5 min. The supernatant was recovered and analyzed. An aliquot of the supernatant was lyophilized. 

### 2.7. Loading of CUR in Micellar SC4OC6 Nanoparticles

A solution of SC4OC6 (10 mg, 0.85 mM) in 10 mL of phosphate buffer (10 mM, neutral pH) was added to solid CUR (15.7 mg). The suspension was vortexed and stirred at 50 °C for 2 h and was left at room temperature for 3 days. The mixture was centrifuged at 6000 rpm for 10 min, then the supernatant was recovered and analyzed. An aliquot of the supernatant was filtered through a 0.2 µm GHP filter (Acrodisc). 

### 2.8. Loading of TMZ in SC4OC6 /CUR Nanoassembly

Solid TMZ (0.1 mg) was added to a colloidal solution of filtered SC4OC6/CUR nanoassembly (1 mg SC4OC6/0.04 mg CUR in 1 mL of phosphate buffer). The mixture was stirred for 1 h at room temperature and was analyzed. An aliquot was lyophilized and was rehydrated by adding pure water, and the sample was analyzed again.

### 2.9. Determination of Loaded TMZ and CUR 

The amount of solubilized TMZ in the presence and absence of SC4OC6 was evaluated by UV–vis spectrophotometry (λ 330 nm) using a calibration curve in 10 mM phosphate buffer at neutral pH. The amount of solubilized CUR in the absence and in the presence of SC4OC6 was determined by a calibration curve in 10 mM phosphate buffer /EtOH (1:1, *v*:*v*). For quantitative analysis, 50 μL of sample, collected before and after filtration, were diluted with 350 μL buffer solution and 400 μL EtOH, and the amount of CUR in solution was measured by absorption at 427 nm. 

The total solubility enhancement of TMZ and CUR was expressed as the solubility enhancement factor (*δ*) calculated by the following [37] equation:

$$\delta =\frac{S-S_{0}}{S_{0}}\times 100$$

where *S*_0_ and *S* denote drug solubility in the absence and presence of SC4OC6, respectively.

### 2.10. CUR Loading Capacity% (LC%) 

The LC% was calculated applying the following formula:

$$\mathrm{LC}\;\left(\%\right)=\frac{\mathrm{mass}\;\mathrm{of}\;\mathrm{CUR}}{\mathrm{mass}\;\mathrm{of}\;\mathrm{CUR}\;\mathrm{loaded}\;\mathrm{SC}4\mathrm{OC}6\;\mathrm{nanoparticles}\;}\times 100$$

### 2.11. TMZ and CUR Stability 

The stability of CUR and TMZ in SC4OC6-based nanoaggregates over time was monitored by UV−vis spectra recorded every 15 min for 12 h in samples incubated at 37 °C. The stability of free CUR and free TMZ was also monitored as reference data. The first order degradation rates were estimated together with the half-life values [38]. 

The stability of TMZ in the SC4OC6 nanoaggregates compared to free TMZ was also monitored by dissolving the lyophilized samples in 0.4 mL of D_2_O. The samples were incubated at 37 °C, and the ^1^H NMR spectra were recorded over time.

## 3. Results and Discussion

### 3.1. Preparation and Characterization of the Micellar SC4OC6 Nanoassembly

The amphiphilic *p*-sulfonato-calix[4]arene bearing hexyl chains tethered at the phenolic OH groups of the calix[4]arene skeleton (SC4OC6, Figure 2b) was synthesized as reported in the literature [32] and was characterized by ^1^H NMR spectroscopy. 

**Figure 3:**
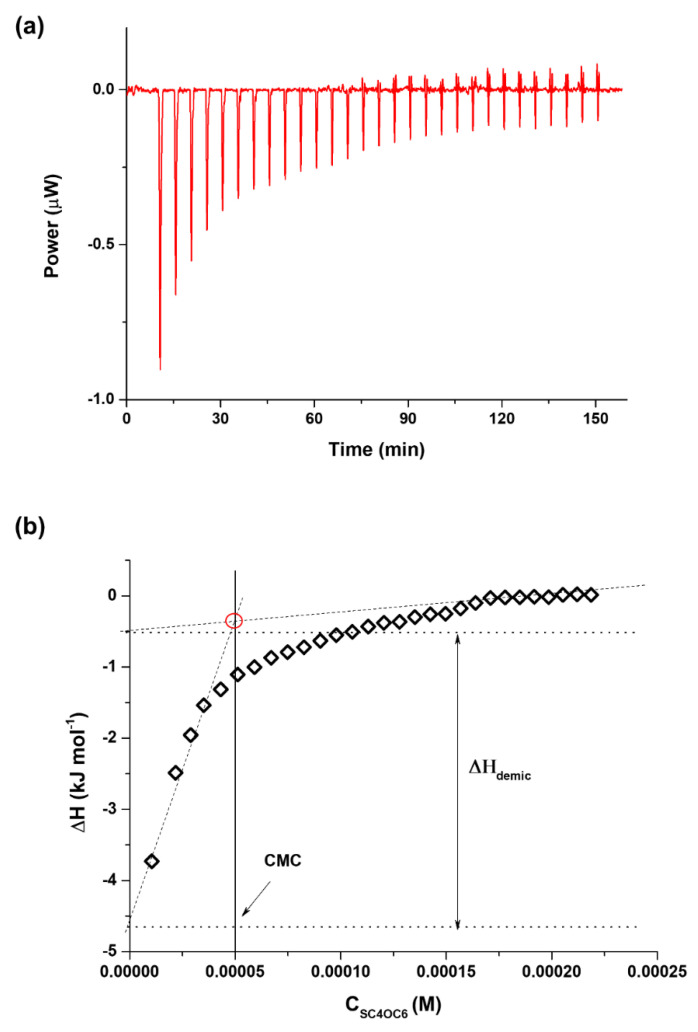
(**a**) ITC titration of SC4OC6 1 mM in 10 mM phosphate buffer at 25 °C; (**b**) reaction enthalpy as a function of the total surfactant concentration (C_SC4OC6_) in the calorimetric cell.

The cone-shaped conformation and the nature of the ionic headgroups of the amphiphilic calix[4]arene derivative are appropriate for high-curvature aggregation in stable micelles [39]. Basilio et al. demonstrated that the amphiphilic SC4OC6 in plain water forms micellar nanoparticles with diameters of 4.27 nm at a critical micellar concentration (CMC) of 0.49 mM [40,41,42]. However, since the present study was conducted at neutral pH (phosphate buffer) to simulate biological conditions, we investigated the aggregation features of SC4OC6 in 10 mM phosphate buffer (I = 20 mM) through ITC measurements. Figure 3a shows an experimental ITC curve obtained for the dilution of a concentrated solution of SC4OC6 (1 mM) into phosphate buffer at 25 °C. Each injection produced an endothermic heat effect which gradually decreased with the number of injections, as typically observed for de-micellization experiments. From the integration of the calorimetric peaks in Figure 3a, the reaction enthalpy as a function of total surfactant concentration in the reaction cell (C_SC4OC6_) was obtained (Figure 3b). 

The calorimetric curve did not show clear sigmoidal features; thus, a linear fit of the datasets in the lower and upper concentration domain had to be performed, as suggested by different authors [43,44]. The intercept of the two straight lines was then determined, with the difference between the two intercepts yielding the value of the enthalpy of micellization. The CMC value was estimated by taking the concentration value at the intersection point of the linear fits at the lower and upper concentration domains of the enthalpogram [45,46]. The thermodynamic parameters obtained are reported in Table S1 along with the CMC and ∆H values determined in plain water at 25 °C (Figure S1) for the sake of comparison.

In 10 mM phosphate buffer (I = 20 mM), the CMC of SC4OC6 resulted significantly lower (0.05 mM) if compared to that found in plain water (0.49 mM), as expected for amphiphilic molecules in the presence of salts [47,48]. Indeed, it has been widely reported that increasing the ionic strength induces a decrease of the CMC value, e.g., micelle formation occurs at a lower total surfactant concentration [49,50,51,52]. The lower CMC value is due to the decrease of the surface charge of the micelles caused by the counterion adsorption that makes the overall self-aggregation process more favored, despite the reduction in enthalpy, than in buffer-free aqueous solutions [53,54,55,56]. Dynamic light scattering analysis showed the presence of nanosized aggregates formed by SC4OC6 in 10 mM phosphate buffer, with a mean hydrodynamic diameter of around 4 nm (volume% distribution mode) (Figure S2).

### 3.2. Preparation and Characterization of the SC4OC6/CUR Nanoassembly

The entrapment of CUR in the SC4OC6 micellar nanoparticles was performed by the simple stirring of solid CUR in the phosphate-buffered colloidal solution of SC4OC6 (at neutral pH) followed by centrifugation to remove the unentrapped drug. A limpid yellow solution was obtained (Figure S3).

#### 3.2.1. Solubility of CUR in the Presence of Micellar SC4OC6 Nanoparticle 

The low solubility of CUR in aqueous medium is a factor that limits the medical application of this multitarget natural drug. 

The capability of SC4OC6 to enhance the solubility of CUR in phosphate buffer at neutral pH, that is immediately evident to the naked eye by the yellow color of the colloidal solution compared to the sample of CUR alone (Figure S3), was confirmed by the absorption of CUR at 430 nm in the UV-vis spectra. Quantitative analyses showed that SC4OC6 enhanced the solubility of CUR by 16 times, with a solubility enhancement factor (δ) of 1514%. The δ value reduced to 770% when the sample passed through a 0.2 µm filter; this could be attributed to the removal of large aggregates of suspended CUR and/or CUR adsorbed on the SC4OC6 nanoaggregate surface. 

Since it has been reported that CUR can be hosted in the cavity of the monomeric SC4OH receptor [57], the formation of an inclusion complex upon SC4OC6/CUR interaction cannot be definitively ruled out. However, the higher enhancement of CUR solubility promoted by the micellar SC4OC6 compared to SC4OH (δ is 216% in water at pH 3 [57]) strongly supports the involvement of the supramolecular nanoaggregate in CUR solubilization. The aromatic rings and the alkyl chains of SC4OC6 packed together provide a hydrophobic environment that favor attractive interactions with the CUR aromatic moieties. The maximum absorption of CUR at 430 nm, which is typical for CUR in surfactant solutions [58], also agreed with CUR entrapped in the SC4OC6 nanoaggregate.

From the value of solubilized CUR, the CUR loading capacity (%) of SC4OC6 at a neutral pH was calculated to be 7%.

#### 3.2.2. Stability of CUR in the SC4OC6 Nanoassembly 

**Figure 4:**
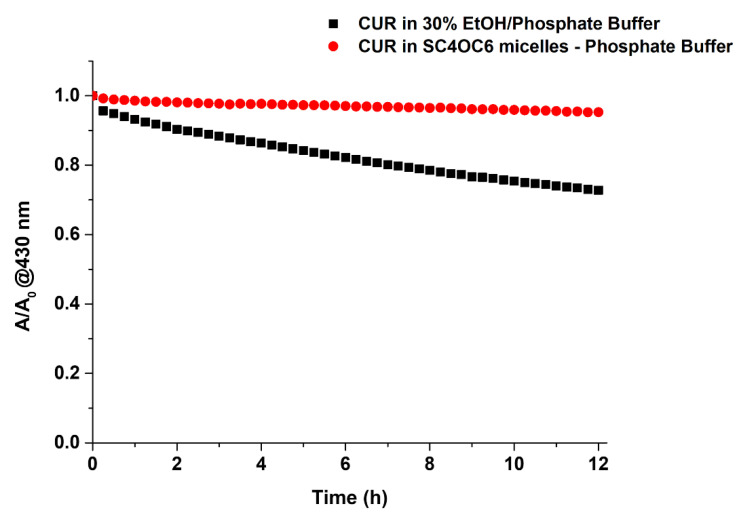
Absorbance values of CUR (λ 430 nm) recorded over time at 37 °C and neutral pH: CUR alone in 30% ethanol/phosphate buffer (0.014 mM, black squares) and CUR (0.014 mM) in SC4OC6 (0.11 mM) nanoparticle in phosphate buffer (red circles).

Low stability in aqueous medium is another factor affecting the medicinal efficacy of CUR. Monitoring the UV-vis absorption of CUR over time, we observed that the entrapment in the SC4OC6 nanocontainer preserved CUR from rapid degradation (Figure S4). The SC4OC6/CUR colloidal solution was incubated at 37 °C for 12 h and was compared with a solution of CUR alone in phosphate buffer containing 30% ethanol (Figure S5). Despite the presence of this solvent, which was used to solubilize and reduce the degradation of CUR in aqueous medium, free CUR at 37 °C degraded faster than CUR loaded in the SC4OC6 nanoaggregate (Figure 4). In the presence of SC4OC6, more than 72% of CUR was maintained after 12 h at 37 °C, and half-life values of 23.5 and 154 h were estimated for the free and entrapped CUR, respectively (Table S2).

#### 3.2.3. Dimensional Analysis and Zeta Potential of the SC4OC6/CUR Nanoassembly

Dynamic light scattering analysis of the SC4OC6/CUR colloidal solution showed the presence of nanoaggregates with mean hydrodynamic diameter around 44 nm (volume% distribution mode) and a polydispersity index (PDI) of 0.23 (Figure S6) after 0.2 µm filtration of the sample. The SC4OC6/CUR nanoassemblies showed a negatively charged surface with zeta potential of −63.3 mV (Figure S7), which was measured by electrophoretic light scattering.

### 3.3. Preparation and Characterization of the SC4OC6/TMZ Nanoassembly 

The loading of TMZ in the micellar SC4OC6 was performed by stirring solid TMZ in a phosphate-buffered solution of SC4OC6 at neutral pH. The removal of the insoluble drug by centrifugation produced a clear colloidal solution. 

#### 3.3.1. Solubility of TMZ in the Presence of the Micellar SC4OC6 Nanoparticle

UV-vis spectrophotometric quantitative analyses (λ 330 nm) showed that the micellar SC4OC6 enhanced the intrinsic TMZ solubility (3.5 mg/mL) with an enhancement solubility factor (δ) of 21% (Figure S8). A similar enhancement was reported for the inclusion complex of the TMZ and cyclodextrins [14]. In the colloidal solution, the amount of soluble TMZ was calculated to be 0.11 mg per mg of SC4OC6. 

#### 3.3.2. Characterization of SC4OC6/TMZ Inclusion Complex 

In principle, multiple binding mechanisms might drive the interaction between TMZ and the micellar SC4OC6. The drug could be entrapped within the nanocontainer architecture or hosted in the cavity of the calix[4]arene macrocycle to form an inclusion complex; our data clearly showed that TMZ is included in the cavity of the SC4OC6 building blocks. 

Generally, the entrapment of a drug into a micelle determines a shift of the absorption band due to its transfer from the polar aqueous phase to the relatively apolar micellar core. Similar to what has been previously reported for the inclusion complexes of TMZ with other macrocycles such as cyclodextrins and cucurbiturils [14,15,16], no shift of the UV-vis absorption of TMZ was observed in the presence of SC4OC6. The formation of an inclusion complex was also suggested by the amount of solubilized TMZ, which is very similar to the one solubilized by a monomeric cyclodextrin [14] and SC4OH [17].

**Figure 5:**
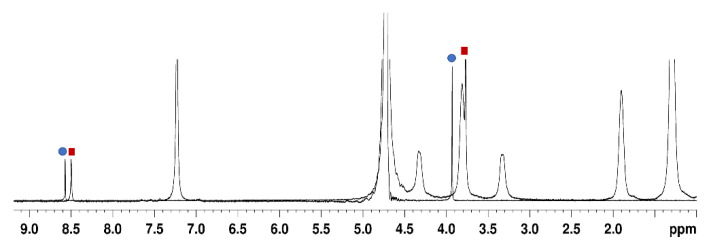
Overlapped ^1^H NMR spectra (D_2_O, phosphate buffer, 297 K) of TMZ alone (5.3 mM, blue circles) and TMZ complexed with 3.5 mM SC4OC6 (5.3 mM, red squares).

The existence of a host–guest inclusion complex was clearly established by NMR spectra. In the presence of SC4OC6, the ^1^H-NMR spectrum of TMZ showed an upfield shift of the aromatic (Δδ 0.03 ppm) and methyl (Δδ 0.06 ppm) protons of the drug (Figure 5). The upfield shift of the TMZ resonances enhanced by increasing the concentration of SC4OC6 (Δδ 0.4 ppm for the methyl group at SC4OC6/TMZ molar ratio 7.5:1). The larger shift of the methyl group resonance indicated that the methyl of the imidazotetrazine ring of TMZ is accommodated in the calix[4]arene cavity. 

The presence of only a resonance peak for the free and complexed TMZ in the proton spectrum of SC4OC6/TMZ (Figure 5) provided evidence that the exchange between free and complexed species is faster than the NMR timescale. 

The upfield shift of the TMZ protons was retained after the lyophilization of the sample, providing evidence that the stability of the complex to the freeze-dry process (Figure S9). This is relevant for the storage and dispensing of orally administrated pharmaceutical ingredients such as TMZ.

#### 3.3.3. Dimensional Analysis and Zeta Potential of the SC4OC6/TMZ Complex

Dynamic light scattering measurements showed that the SC4OC6/TMZ colloidal solution contains nanoaggregates with diameters of around 4 nm (volume% distribution mode) and a PDI of 0.5 (Figure S10). 

Electrophoretic light scattering showed that the SC4OC6/TMZ nanoassemblies possess an average zeta potential of –36.3 ± 22 mV (65% population with −39.5 ± 9.35 and 12% with −78.6 ± 4.63). The negative zeta potential corroborated the hypothesis that the calixarene cavities decorated with the sulfonato groups are exposed on the surface of the nanoparticles and are available for host–guest interactions with TMZ. 

#### 3.3.4. Stability of TMZ when Complexed with SC4OC6

**Figure 6:**
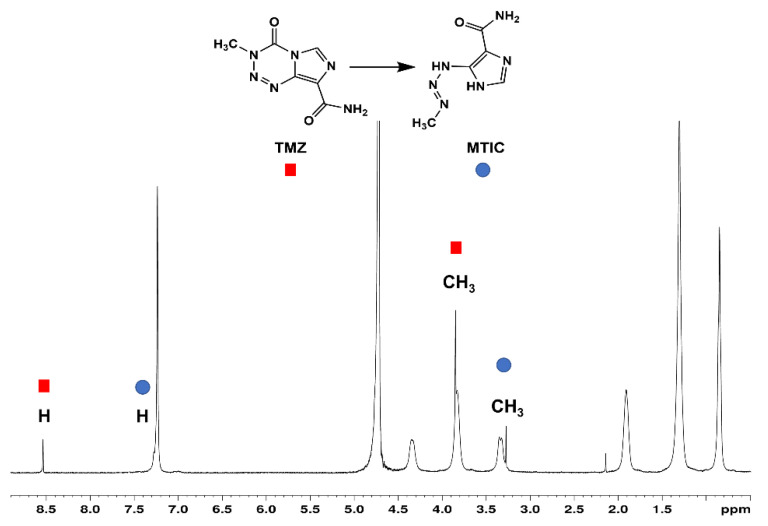
^1^H NMR spectra of SC4OC6/TMZ (7.4 mM/4.5 mM) after incubation for 6 h at 37 °C. TMZ signals (red square) and MTIC signals (blue circle).

The ability of SC4OC6 to protect TMZ from rapid degradation at neutral pH was investigated along with a control solution of TMZ alone. Heating the SC4OC6/TMZ complex at 37 °C yielded the appearance of ^1^H-NMR resonances at 7.23 ppm and 3.27 ppm, which is indicative of the degradation of TMZ to MTIC (Figure 6). However, the relative resonance areas showed a lower degradation of TMZ after 6 h incubation at 37 °C (Figure 6) compared to that of free TMZ [17].

The effect of the SC4OC6 nanoassembly in preserving TMZ from rapid degradation was also investigated by monitoring the decrease of the absorption band at 330 nm of TMZ at 37 °C for 12 hours when compared with that of free TMZ. A slight improvement of the TMZ half-life from 5.8 to 6.3 hours was observed in the presence of SC4OC6 (Table S3, Figures S11 and S12). 

### 3.4. Co-Entrapment of TMZ and CUR in the SC4OC6 Nanocontainer

The SC4OC6/CUR/TMZ ternary nanoassembly was prepared by adding TMZ (10% with respect to the amount of SC4OC6) to the colloidal filtered solution of SC4OC6/CUR. After stirring, a clear and yellow colloidal solution was obtained. 

NMR spectra showed that the SC4OC6 loaded with CUR maintained the capability to complex TMZ, as evidenced by the upfield shift of the TMZ protons (Figure S13). The ternary nanoassembly was stable to freeze-dry, as confirmed by the proton NMR spectrum of the suspended lyophilized powder (Figure S13), thus emphasizing the potential application of this system for the simultaneous formulation and oral administration of CUR and TMZ. 

Based on literature data, the amounts of CUR (40 μg/mL) and TMZ (100 μg/mL) entrapped in the SC4OC6 nanoaggregate (1 mg/mL) are enough to investigate the synergistic effect of TMZ/CUR against glioblastoma. As an example, a synergistic effect was demonstrated on U87MG glioblastoma cells through the administration of TMZ and CUR at the concentrations of 15.63 μg/mL and 1.25 μg/mL, respectively [4]. 

#### Dimensional and Zeta Potential Analysis of SC4OC6/CUR/TMZ 

**Figure 7:**
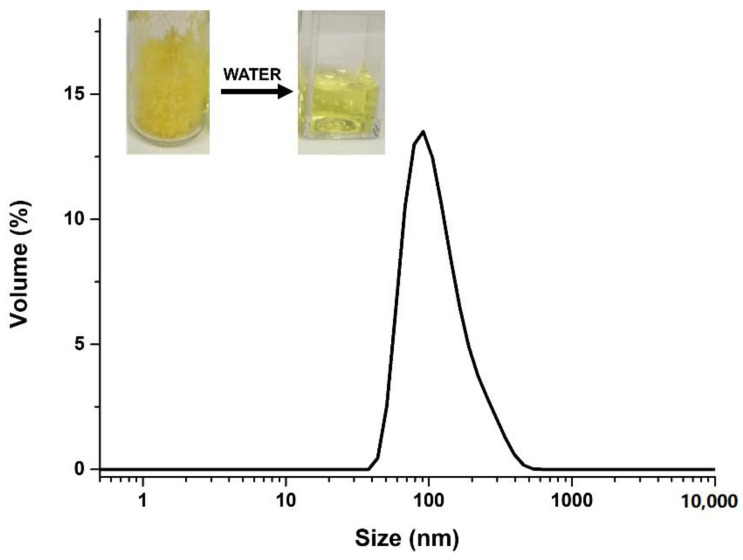
Volume-weighted hydrodynamic diameter distribution of the SC4OC6/CUR/TMZ (0.85 mM/0.11 mM/0.5 mM) ternary assembly and pictures of the lyophilized powder re-suspended by water addition.

Dynamic light scattering measurements performed on the SC4OC6/CUR/TMZ colloidal solution showed the presence of nanoaggregates with hydrodynamic diameters around 122 nm in the colloidal solution and PDI of 0.26 (Figure 7). No significant variation of the zeta potential value (−64.6 mV) compared to that of the SC4OC6/CUR assembly was observed after TMZ complexation (Figure S14).

### 3.5. Solution Thermodynamics of the SC4OC6/TMZ and SC4OC6/CUR/TMZ Complexes Formation 

For a more in-depth investigation of the interactions of TMZ with the micellar SC4OC6 nanoparticle and the determination of the binding parameters and driving forces of the molecular recognition processes in solution we performed calorimetric studies. 

ITC measurements were also conducted to establish whether TMZ was able to interact with the amphiphilic calixarene SC4OC6 in either its aggregated or monomeric form as well as in the presence or absence of loaded CUR. 

The thermodynamic parameters associated with the guest–micelle interaction contributed to the establishment of the positioning of the guest/drug within the micellar aggregate. The guest can be completely included within the hydrophobic core of the micelle due to the interaction with the bulkier lipophilic chains, which may penetrate up to a certain depth of the micellar palisade layer or may be somehow adsorbed on the micellar surface. 

**Figure 8:**
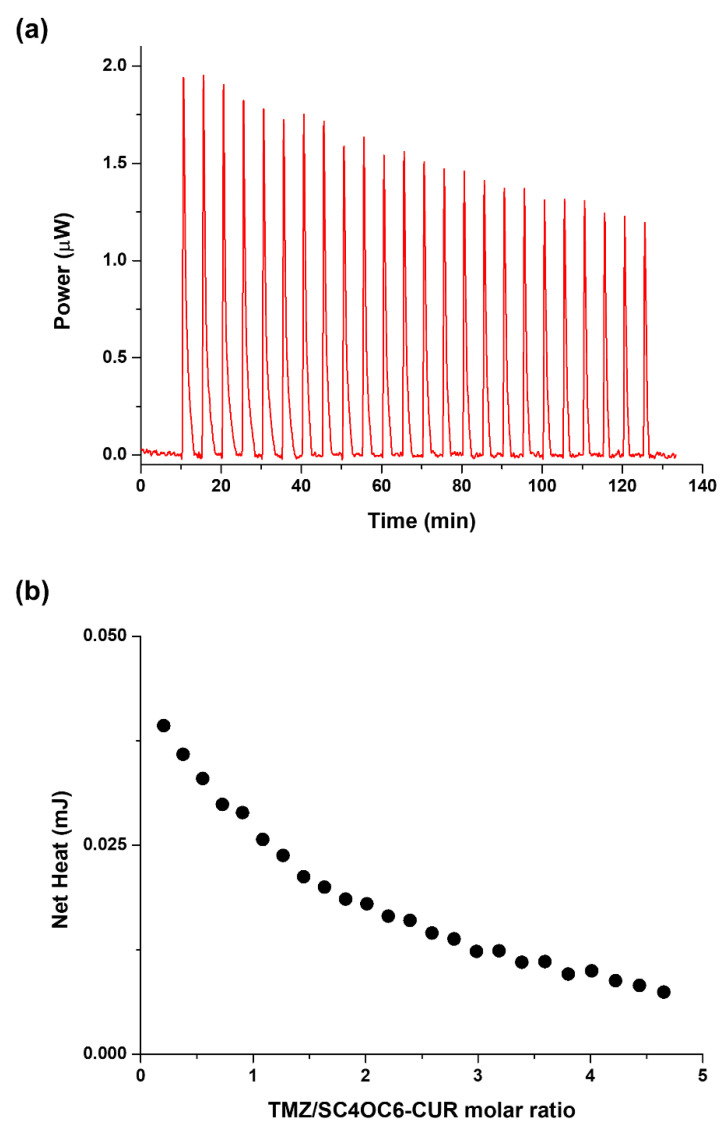
(**a**) ITC titration of TMZ 5 mM into SC4OC6/CUR nanoassembly 0.3 mM (above CMC) at 25 °C in neutral solution (phosphate buffer); (**b**) integrated heat data.

First, calorimetric experiments were run by titrating solutions of TMZ into SC4OC6 or SC4OC6/CUR solutions at experimental conditions that are able to guarantee the presence of the micellar aggregates in the calorimetric vessel. A typical ITC titration for the SC4OC6/CUR/TMZ nanoassembly in a neutral solution at 25 °C is shown in Figure 8. An example of an ITC titration curve for the SC4OC6/TMZ complex is shown in Figure S15 together with a typical blank experiment (Figure S16).

The calorimetric curves show that the gross reaction heat released when the TMZ is titrated into a micellar calixarene solution both in the presence and in the absence of curcumin is larger than the heat from the blank experiment, resulting in remarkable net heat values. Thus, the thermodynamic parameters were determined by analyzing the pattern obtained from the integrated heat data.

To assess the role played by the micellar nanoassembly in the drug recognition process, ITC measurements were also conducted by titrating the TMZ into a SC4OC6 solution at a concentration below CMC (0.01–0.02 mM), i.e., when the calixarene is in its monomeric form. The overlap between blank and titration experiments (Figure S17) together with the negligible net heat values recorded (Figure S18) unambiguously revealed that, at this concentration, SC4OC6 is not able to establish any significant interaction with TMZ. 

However, since the SC4OC6 concentration employed in this latter set of measurements is very small (due to the low CMC value of SC4OC6), to assess whether TMZ is included in the calixarene cavity, as observed via NMR experiments, ITC titrations were also conducted using SC4OH, a *p*-sulfonato-calix[4]arene that is analogous to SC4OC6 but that does not have alkyl chains at the lower rim. SC4OH is not able to self-aggregate, thus allowing the use of larger concentrations and experimental conditions similar to those employed for the micellar aggregates. A suitable calorimetric pattern was obtained (Figure S19), which enabled the determination of the binding parameters for the inclusion of TMZ into the SC4OH cavity. 

Data obtained by the ITC measurements were analyzed by assuming the formation of a 1:1 inclusion complex between TMZ and SC4OH; other models were tested but were always rejected by the program. A model that assumes a 1:1 species between the drug and the micellar aggregate formed by SC4OC6 (both in the presence and in the absence of curcumin) was also employed, as previously reported by us [59] and in line with many research groups that used the “one site” binding model for the refinement of the thermodynamic parameters of similar systems [60,61,62,63,64].

**Figure 9:**
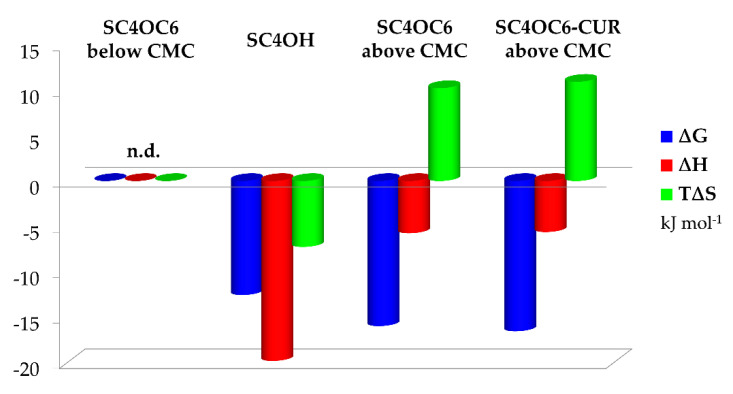
Thermodynamic parameters for the complex formation of TMZ with calixarene-based receptors in their monomeric or aggregated form at 25 °C in neutral aqueous buffered solution.

The binding constants and parameters for the systems that were investigated are reported in Table 1 and shown in Figure 9.

Two main recognition processes have been investigated: the formation of the complex between TMZ and the monomeric SC4OH host as well as the interaction of TMZ with SC4OC6-based micelles, both of which were evaluated in the presence and in the absence of loaded curcumin.

The data reported in Table 1 show no relevant difference in the binding parameters for SC4OC6 and SC4OC6/CUR nanoaggregates, suggesting that the loaded CUR does not affect the recognition process of TMZ in solution. Conversely, a smaller affinity was found with the monomeric SC4OH receptor, revealing that binding is more favored in the presence of nanoaggregates.

The splitting of the Gibbs free binding energy into the enthalpic and entropic contributions enabled factors and forces that cannot be determined by the ΔG value alone to be highlighted [65,66]. The formation of the complex species between TMZ and SC4OH is both enthalpically driven and favored: CH–π and π–π interactions involving the drug and the host cavity drive the complex formation, and these attractive forces override the energy cost needed for the desolvation of the interacting reagents. The recognition process is also entropically unfavored because of the loss of degrees of freedom due to host–guest complex formation, which prevails over host and guest desolvation [67,68,69].

A totally different picture appears when dealing with the micellar aggregates. The interaction of TMZ with SC4OC6-based micelles both in the presence and in the absence of loaded CUR is both an entropy and enthalpy favored process, with entropy being the main driving force (|ΔH| < |TΔS|). This favorable entropic contribution is due to the desolvation of both the guest and the external layer of the micelle surface upon guest binding. Indeed, TMZ weakly interacts with the exterior micelle surface and should not be able to be inserted into the palisade layer of the aggregate. This interaction causes the release of water of hydration to the bulk solvent (large and positive entropy values). The favorable ΔH value is probably due to attractive forces between the calixarene hydrophilic heads and the TMZ backbone as well as to “frustrated” water molecules leaving the micelle core and creating new hydrogen bonding networks with bulk water molecules and the exterior layer of the nanoaggregate [70,71].

### 3.6. Stability of TMZ in the Ternary SC4OC6/CUR/TMZ Assembly 

**Figure 10:**
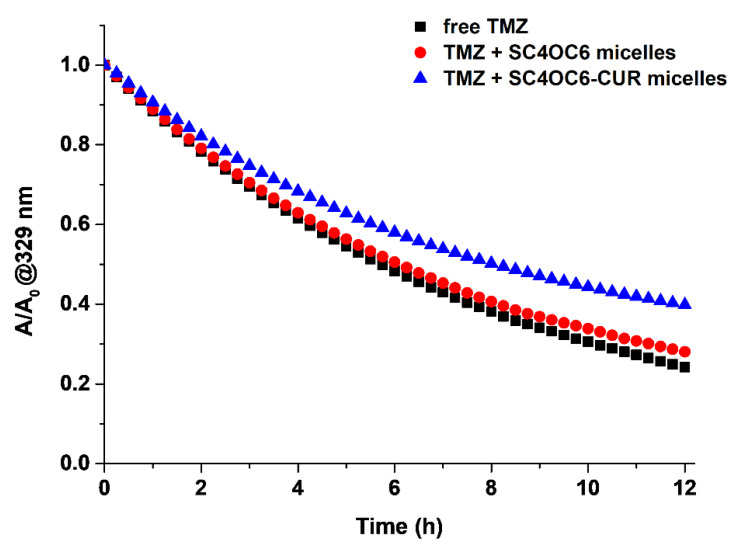
Absorbance values recorded at 330 nm for free TMZ (0.11 mM) (black squares), TMZ (0.11 mM) in SC4OC6 (0.22 mM) complex (red circles) and in SC4OC6/CUR/TMZ nanoassembly (0.22 mM/0.014 mM/0.22 mM) (blue triangles) in neutral aqueous solution at 37 °C over time.

The preservative effect of SC4OC6 on TMZ in the presence of CUR compared to the free drug was investigated by UV-vis spectrophotometry. The UV-vis spectra (Figure S20) showed an enhancement of the TMZ half-life of more than 40% (Table S3) for the SC4OC6/CUR/TMZ nanoassembly incubated at 37 °C for 12 h compared to TMZ alone. Since the binding affinities of TMZ with SC4OC6 and SC4OC6/CUR are comparable, the slower degradation rate of TMZ in the ternary SC4OC6/CUR/TMZ nanossembly (Figure 10), compared to the SC4OC6/TMZ complex, could be attributed to the stabilization effect played by CUR, as reported for this molecule in combination with other anticancer drugs [23]. 

### 3.7. Potential of SC4OC6/CUR/TMZ in Nanocarrier-Based Combined Chemotherapy 

In the research of novel nanocontainers for a combined drug therapy, the micellar SC4OC6 nanoparticle is attractive as it matches, within the same structure, the hydrophobic regions of the micelle and the host–guest recognition sites of the calix[4]arene cavity. The SC4OC6 nanocontainer, in addition to properties such as the co-loading of drugs and the enhancement of TMZ and CUR solubility and stability, possesses further prerequisites for applications as a nanoscale drug delivery system. Examples include: (i) sizes of 70–200 nm are desirable for reaching tumor tissues by exploiting the enhanced permeability and retention (EPR effect) in tumor vasculature [72,73]; (ii) negatively charged nanoparticles can resist protein adsorption and can exhibit prolonged blood circulation time by performing less unspecific cell uptake [74]; (iii) no significant cytotoxicity was reported for the micellar SC4OC6–based nanocarriers [75,76]; and (iv) the amount of TMZ and CUR loaded in the calixarene nanocontainer are satisfactory for investigating their antiglioblastoma and synergistic effect [4]. These features, along with the finding that the inclusion of TMZ in the cavity of SC4OH enhanced the anti-glioblastoma efficacy of TMZ in vitro and in vivo experiments [17], strongly support the potential of SC4OC6 as a novel nanocarrier of TMZ and CUR for combined therapy applications.

## 4. Conclusions

Nowadays, nanoparticles with a variety of structures are actively being investigated and are on the horizon as an alternative strategy for enhancing the safety and efficacy of anticancer treatment. Our findings showed that micellar nanoparticles formed by the spontaneous self-assembly of an amphiphilic *p*-sulfonato-calix[4]arene derivative can effectively load anticancer drugs such as temozolomide and curcumin, both singularly and in combination, in a neutral buffered aqueous solution. The entrapment of the drugs within the calixarene-based nanocontainer enhanced their solubility and reduced their fast degradation in solution. The molecular recognition process is driven by entropy (mainly due to desolvation) and only occurs efficiently when the calixarene receptor is in a nano-aggregated structure. The easy preparation of the nanocarrier, which does not require the use of organic solvents, the improved features of the loaded drugs that may act in a synergistic way, and the known ability of the *p*-sulfonato-calixarene derivatives as drug delivery systems make the investigated nanoparticles promising systems for multi-drug anticancer therapy.

## Figures and Tables

**Table 1:** LogK values and thermodynamic parameters for the interaction of TMZ with calixarene-based receptors in their monomeric or aggregated form at 25 °C in neutral aqueous buffered solution.

	log K	ΔH (kJ mol^−1^)	ΔS (J K^−1^ mol^−1^)
(TMZ)(SC4OC6)_below CMC_	n.d.	n.d.	n.d.
(TMZ)(SC4OC6)_above CMC_	2.8 (1)	−5.75 (6)	34 (2)
(TMZ)(SC4OC6-CUR)_above CMC_	2.9 (1)	−5.62 (4)	36 (2)
(TMZ)(SC4OH)	2.2 (1)	−15.82 (7)	−25 (2)

## Data Availability

Data are available from the corresponding authors upon reasonable request.

## Supplementary Materials

The following are available online at https://www.mdpi.com/article/10.3390/nano11112930/s1: Figure S1: ITC titration of SC4OC6 into plain water, Figure S2: Hydrodynamic diameter distribution of SC4OC6, Figure S3: UV-vis and fluorescence spectra of SC4OC6/CUR, Figure S4: UV-vis spectra of SC4OC6/CUR over time, Figure S5: UV-vis spectra of curcumin over time, Figure S6: Hydrodynamic diameter distribution of SC4OC6/CUR, Figure S7: Zeta potential distribution of SC4OC6/CUR, Figure S8: UV-vis spectra of TMZ and SC4OC6/TMZ, Figure S9: Overlapped 1H NMR spectra of TMZ and SC4OC6/TMZ complex, Figure S10: Hydrodynamic diameter distribution of SC4OC6/TMZ, Figure S11: UV-vis spectra of SC4OC6/TMZ over time, Figure S12: UV-vis spectra of TMZ over time, Figure S13: Overlapped 1H NMR spectra of TMZ and lyophilized SC4OC6/CUR/TMZ, Figure S14: Zeta potential distribution of SC4OC6/CUR/TMZ, Figure S15: ITC titration of TMZ into SC4OC6, Figure S16: Typical “blank” experiment, Figure S17: ITC titration and blank experiments of TMZ into SC4OC6, Figure S18: Net heat curve for the TMZ - SC4OC6 (below CMC) system, Figure S19: ITC titration of TMZ into SC4OH and integrated heat data, Figure S20: UV-vis spectra of SC4OC6/CUR/TMZ over time, Table S1: Thermodynamic parameters for SC4OC6 self-aggregation process, Table S2: Kinetic parameters for curcumin and for SC4OC6/CUR, Table S3: Kinetic parameters for free TMZ, SC4OC6/TMZ and SC4OC6/CUR/TMZ.
