# Supplementary material for: Co-Loading of Temozolomide and Curcumin into a Calix[4]arene-Based Nanocontainer for Potential Combined Chemotherapy: Binding Features, Enhanced Drug Solubility and Stability in Aqueous Medium
Source: Nanomaterials (Basel). 2021 Nov 2;11(11):2930. doi: 10.3390/nano11112930 (PMC8623626; doi:10.3390/nano11112930)
Supplement: Supplementary file 1 [file nanomaterials-11-02930-s001.zip › nanomaterials-1404648-supplementary.pdf]

Supplementary Materials

Co-loading of Temozolomide and Curcumin into a Calix[4]arene-Based Nanocontainer for Potential Combined Chemotherapy: Binding Features, Enhanced Drug Solubility and Stability in Aqueous Medium

Rossella Migliore [1], Nicola D'Antona [1], Carmelo Sgarlata [2,]* and Grazia M. L. Consoli [1,]*

[1] Istituto di Chimica Biomolecolare, Consiglio Nazionale delle Ricerche, Via Paolo Gaifami 18, 95126 Catania, Italy; rossella.migliore@unict.it (R.M.); nicola.dantona@icb.cnr.it (N.D.)
[2] Dipartimento di Scienze Chimiche, Università degli Studi di Catania, Viale Andrea Doria 6, 95125 Catania, Italy
* Correspondence: sgarlata@unict.it (C.S.); grazia.consoli@icb.cnr.it (G.M.L.C.)

Content

Figure S1. ITC titration of SC4OC6 into plain water **S3**

Table S1. Thermodynamic parameters for SC4OC6 self-aggregation process **S4**

Figure S2. Hydrodynamic diameter distribution of SC4OC6 **S4**

Figure S3. UV-vis and fluorescence spectra of SC4OC6/CUR **S5**

Figure S4. UV-vis spectra of SC4OC6/CUR over time **S6**

Figure S5. UV-vis spectra of curcumin over time **S6**

Table S2. Kinetic parameters for curcumin and for SC4OC6/CUR **S7**

Figure S6. Hydrodynamic diameter distribution of SC4OC6/CUR **S7**

Figure S7. Zeta potential distribution of SC4OC6/CUR **S8**

Figure S8. UV-vis spectra of TMZ and SC4OC6/TMZ **S8**

Figure S9. Overlapped ^{1}H NMR spectra of TMZ and SC4OC6/TMZ complex **S9**

Figure S10. Hydrodynamic diameter distribution of SC4OC6/TMZ **S9**

Figure S11. UV-vis spectra of SC4OC6/TMZ over time **S10**

Figure S12. UV-vis spectra of TMZ over time **S10**

Figure S13. Overlapped ^{1}H NMR spectra of TMZ and lyophilized SC4OC6/CUR/TMZ **S11**

Figure S14. Zeta potential distribution of SC4OC6/CUR/TMZ **S11**

Figure S15. ITC titration of TMZ into SC4OC6 **S12**

Figure S16. Typical "blank" experiment **S12**

Figure S17. ITC titration and blank experiments of TMZ into SC4OC6 **S13**

Figure S18. Net heat curve for the TMZ - SC4OC6 (below CMC) system **S13**

Figure S19. ITC titration of TMZ into SC4OH and integrated heat data **S14**

Figure S20. UV-vis spectra of SC4OC6/CUR/TMZ over time **S15**

Table S3. Kinetic parameters for free TMZ, SC4OC6/TMZ and SC4OC6/CUR/TMZ **S16**

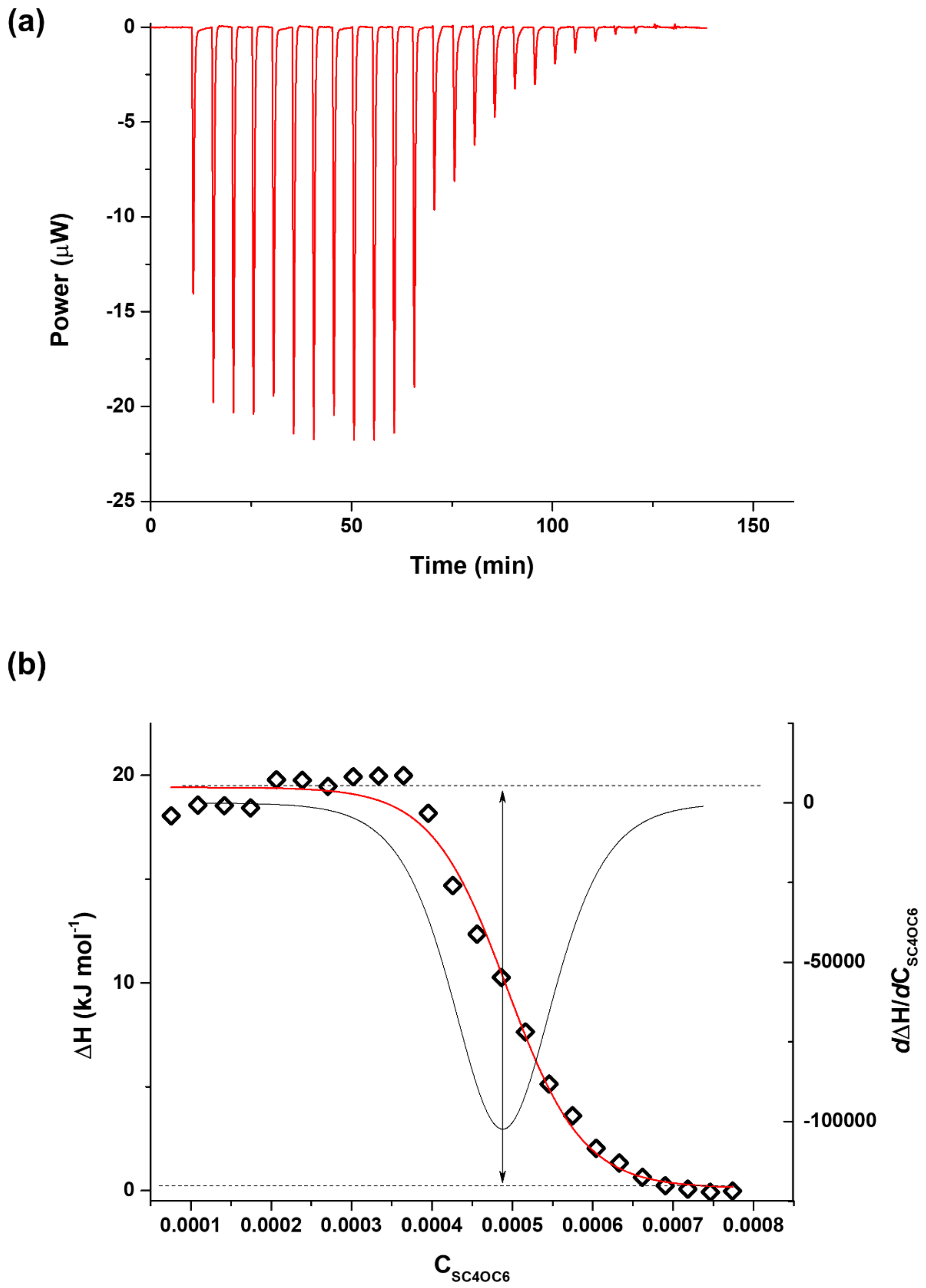

Figure S1. (**a**) ITC titration of SC4OC6 4 mM into plain water at 25 °C; (**b**) reaction enthalpy as a function of the total surfactant concentration (C_{SC4OC6}) in the calorimetric cell.

Table S1. CMC and enthalpy values for SC4OC6 self-aggregation process at 25 °C.

	CMC (mM)	ΔH (kJ mol^{-1})
Plain water	0.492 (5)	-21.7 (2)
Phosphate buffer (I = 20 mM)	0.05 (1)	-4.8 (2)

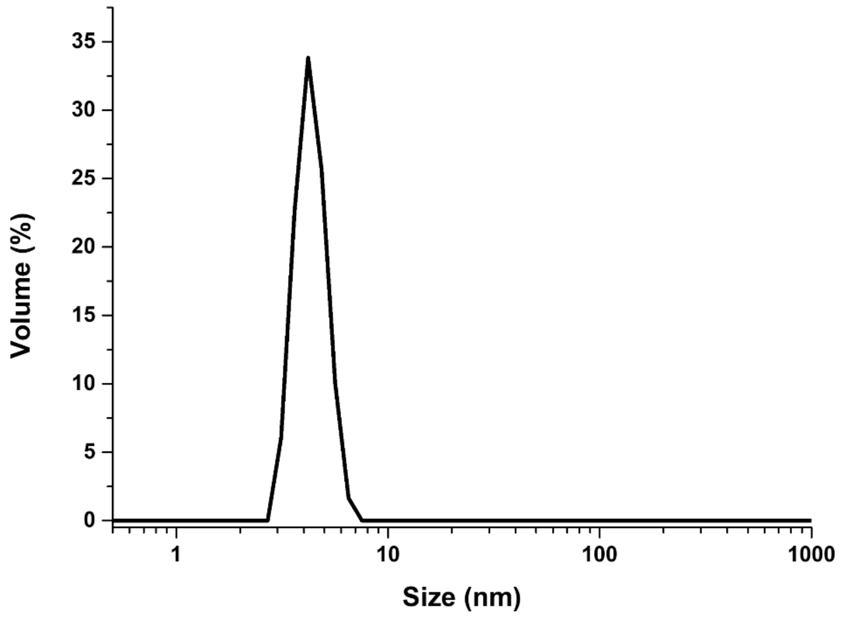

Figure S2. Volume weighted hydrodynamic diameter distribution of SC4OC6 (1.7 mM) in 10 mM phosphate buffer.

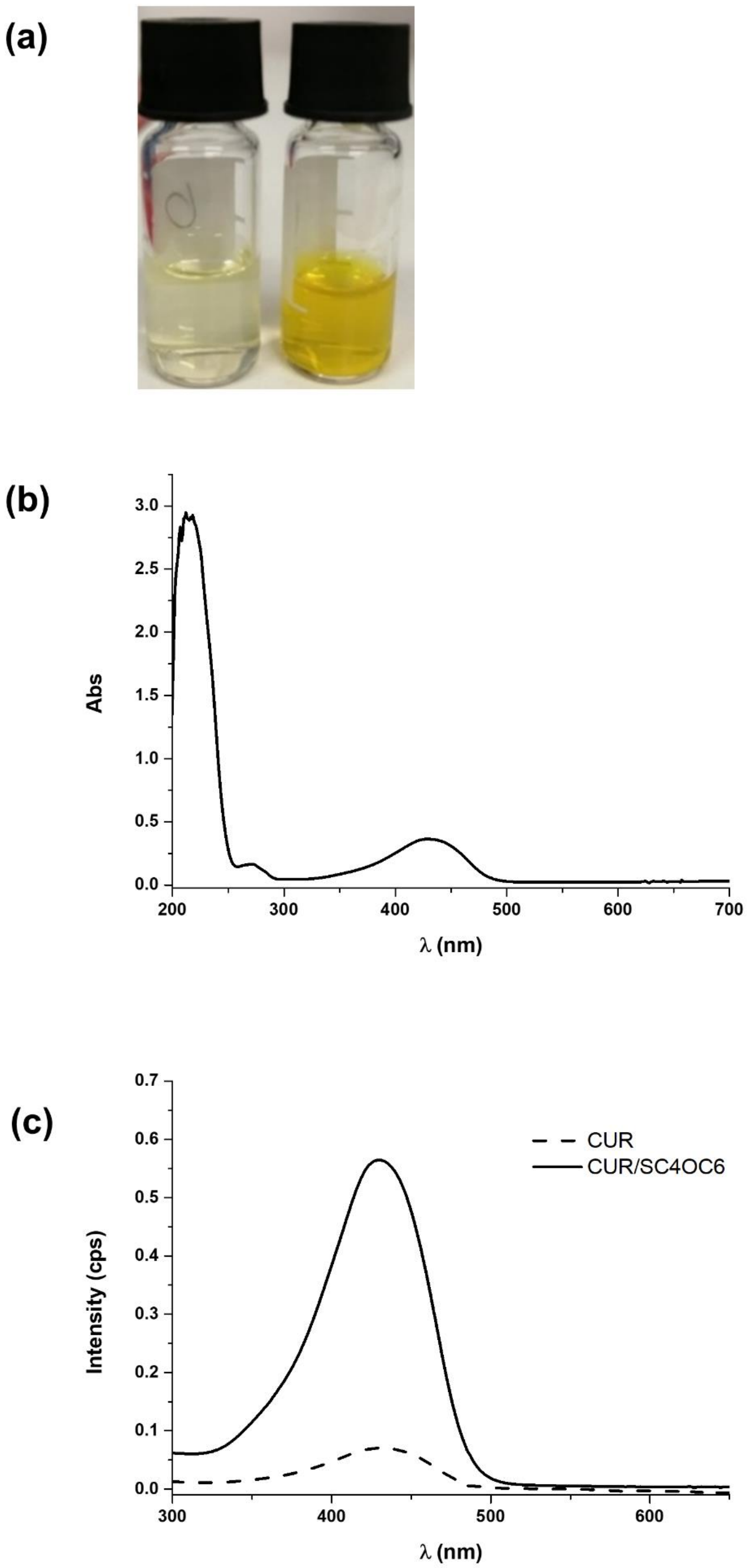

Figure S3. (**a**) Picture of the colloidal solution of CUR alone (right) and with SC4OC6 (left) in 10 mM phosphate buffer; (**b**) UV-vis spectrum of SC4OC6/CUR (0.091 mM/0.012 mM); (**c**) fluorescence spectra of CUR alone (0.0016 mM, dashed line) and CUR (0.012 mM, solid line) in the presence of SC4OC6 in 10 mM phosphate buffer.

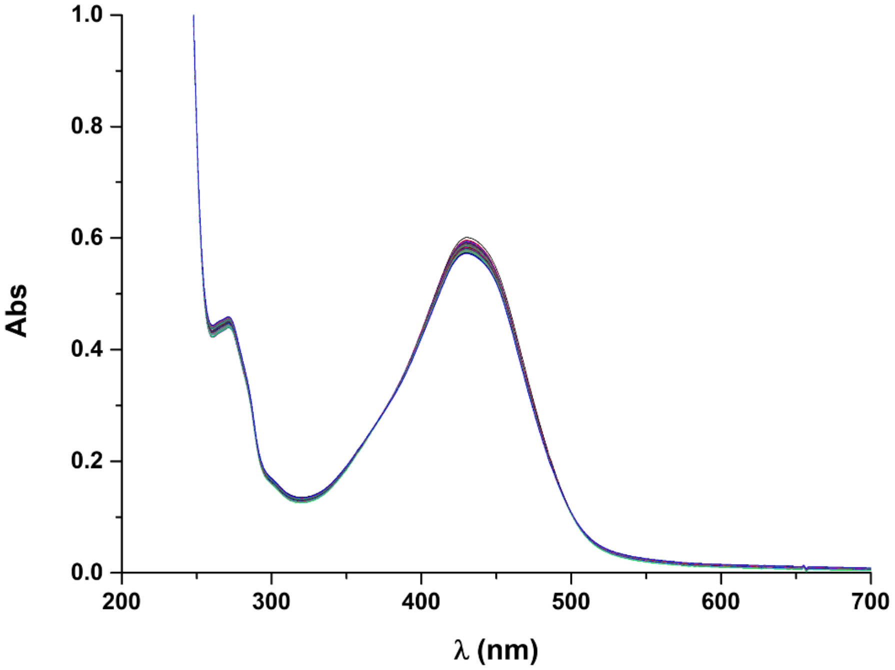

Figure S4. UV-vis spectra of SC4OC6/CUR (0.11 and 0.014 mM respectively) in 10 mM phosphate buffer recorded every 15 minutes at 37 °C.

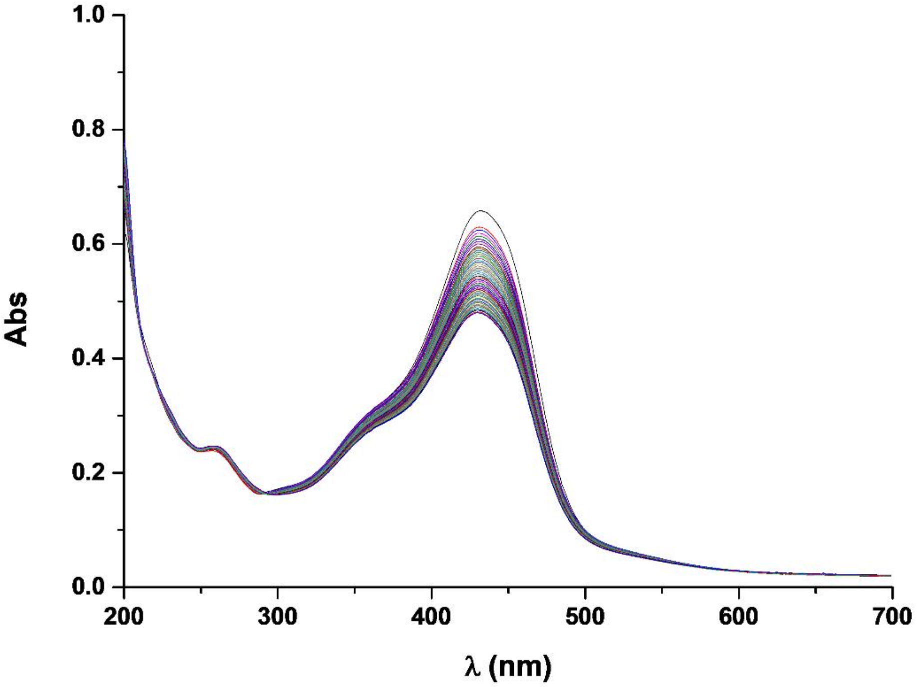

Figure S5. UV-vis spectra of curcumin (0.014 mM) in 30% ethanol/phosphate buffer recorded every 15 minutes at 37 °C.

Table S2. First order degradation rate constant and half-life values for curcumin in 30% ethanol/phosphate buffer and for SC4OC6/CUR in 10 mM phosphate buffer at 37 °C.

	**k** **(min^{-1})**	**$t_{1/2}$** **(h)**
Free CUR	0.00049 (1)	23.5
CUR in SC4OC6 micelles	0.000075 (2)	154

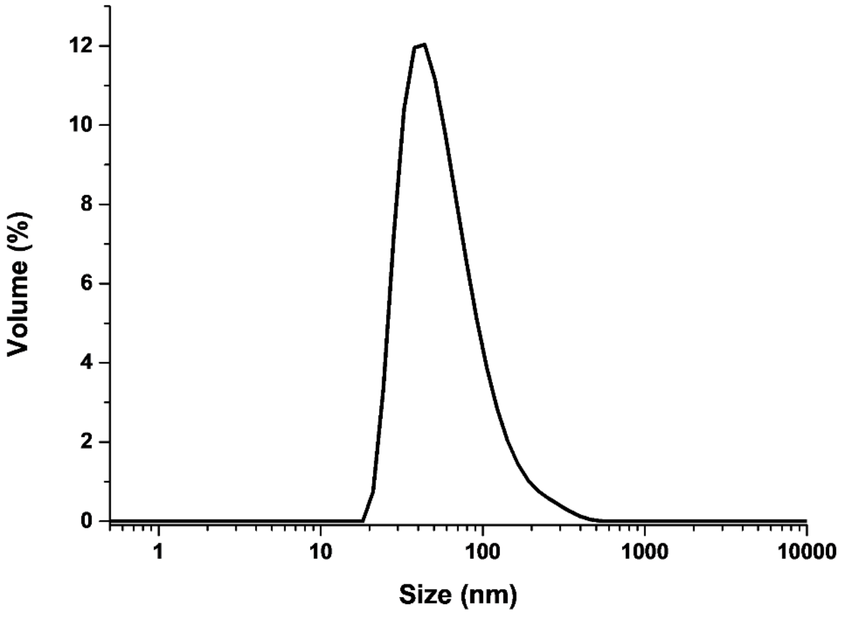

Figure S6. Volume weighted hydrodynamic diameter distribution of SC4OC6/CUR (0.85 mM/0.11 mM) in 10 mM phosphate buffer.

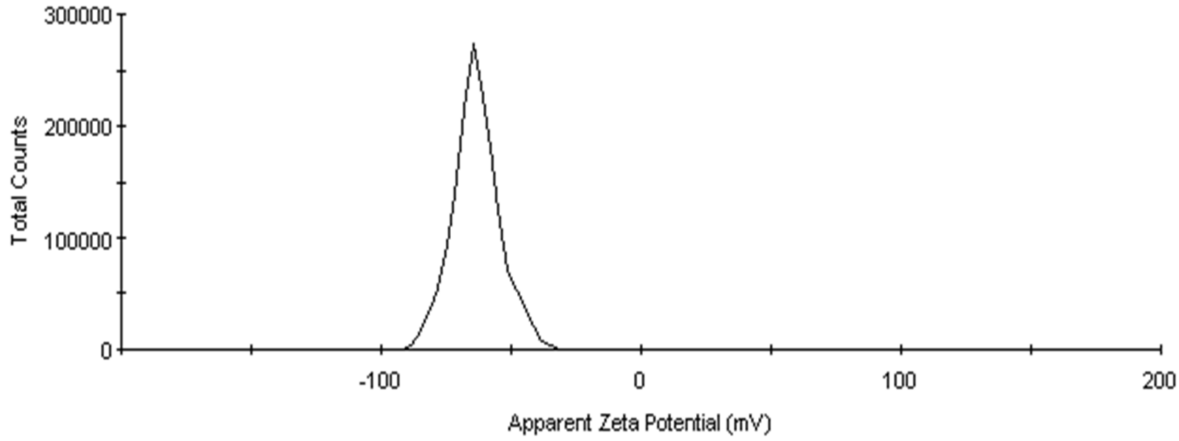

Figure S7. Zeta potential distribution of SC4OC6/CUR (0.85 mM/0.11 mM) in 10 mM phosphate buffer.

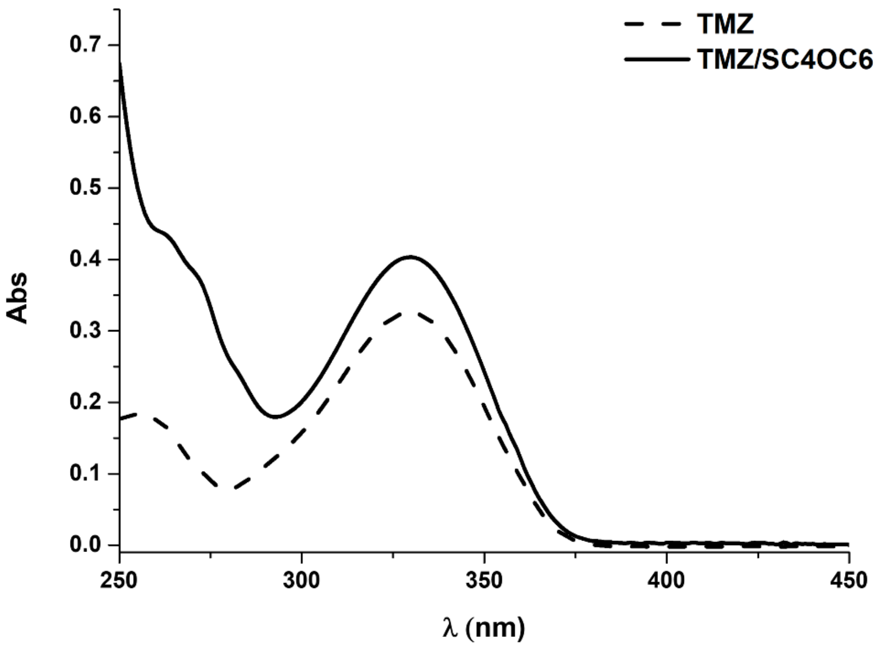

Figure S8. UV-vis spectra of TMZ alone (0.03 mM, dashed line) and TMZ (0.0365 mM) in the presence of SC4OC6 (solid line) in 10 mM phosphate buffer.

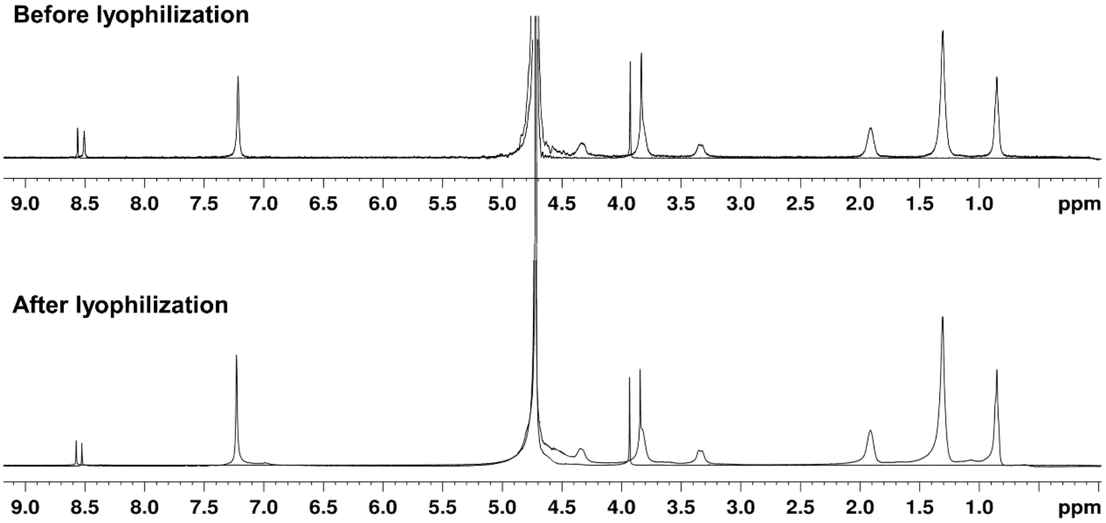

Figure S9. Overlapped ^{1}H NMR spectra (D_2O, phosphate buffer, 297 K) of TMZ (4.5 mM) and SC4OC6/TMZ (7.4 mM/4.5 mM) complex before (up) and after (bottom) freeze-drying.

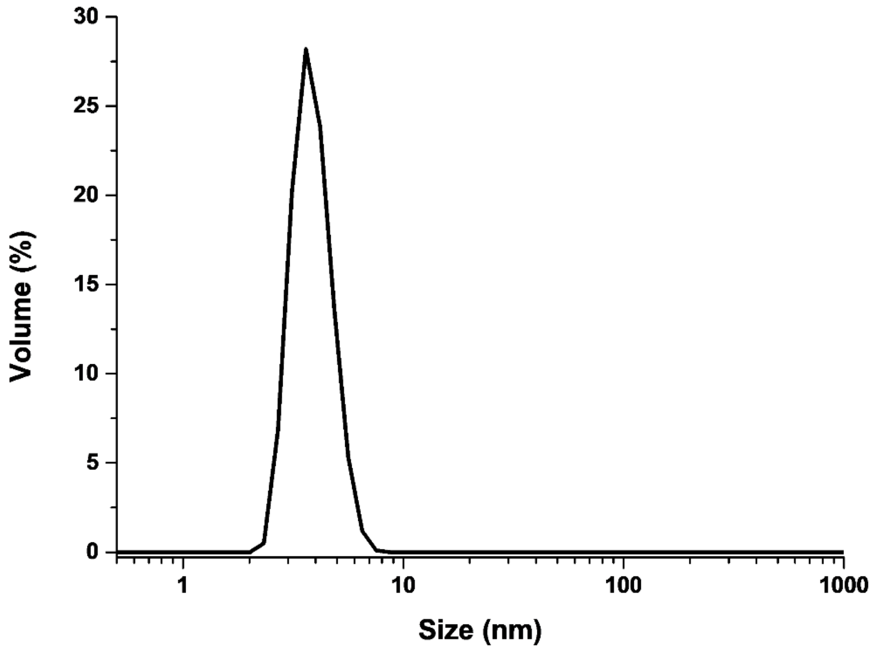

Figure S10. Volume weighted hydrodynamic diameter distribution of SC4OC6/TMZ (1.7 mM/1.03 mM) complex in 10 mM phosphate buffer.

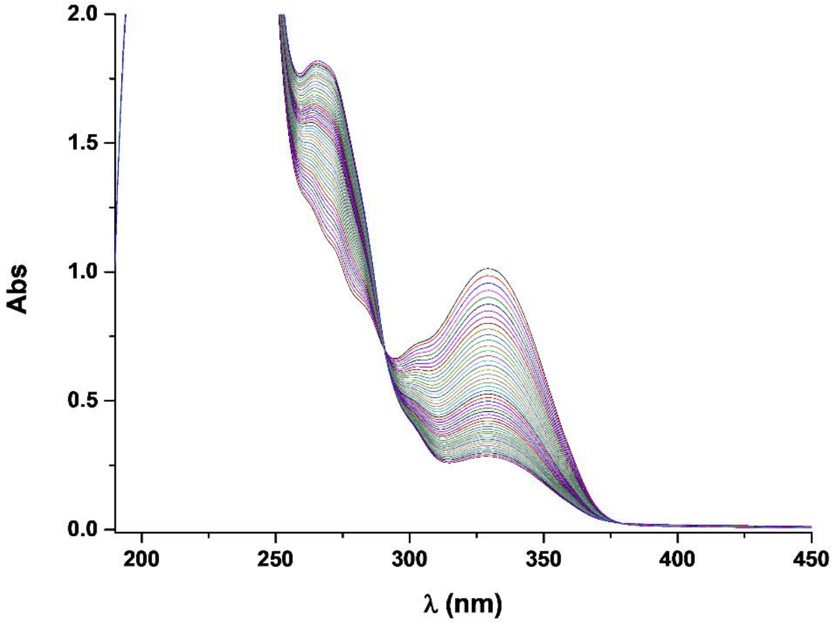

Figure S11. UV-vis spectra of SC4OC6/TMZ (0.2 and 0.1 mM respectively) in 10 mM phosphate buffer recorded every 15 minutes at 37 °C.

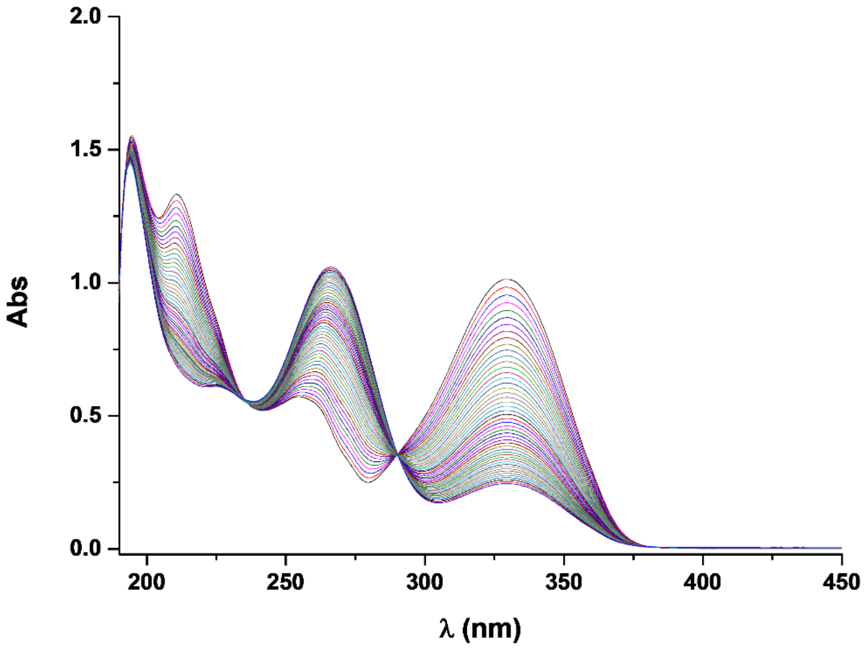

Figure S12. UV-vis spectra of TMZ (0.1 mM) in 10 mM phosphate buffer recorded every 15 minutes at 37 °C.

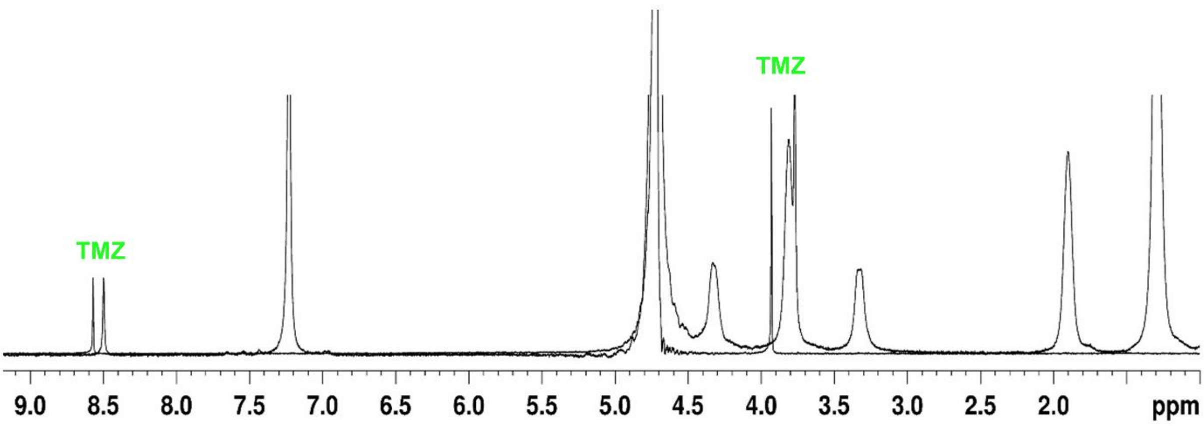

Figure S13. Overlapped ^{1}H NMR spectra (D_2O, phosphate buffer, 297 K) of TMZ (4.5 mM) and lyophilized SC4OC6/CUR/TMZ (7.4 mM/0.95 mM/4.6 mM).

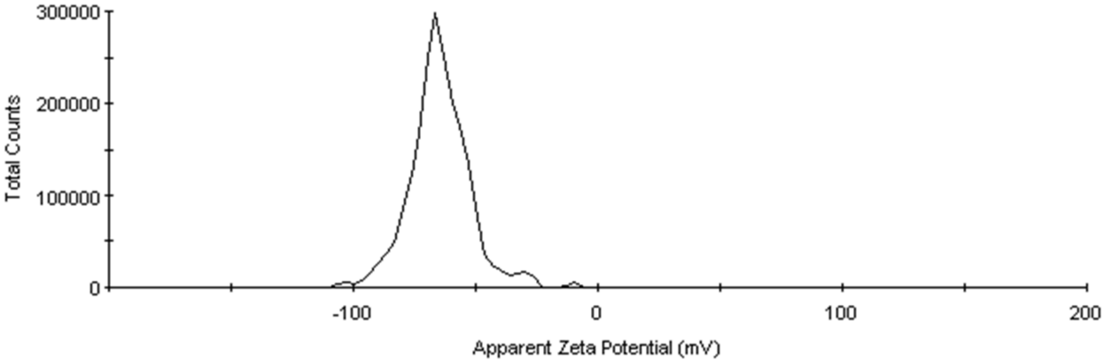

Figure S14. Zeta potential distribution of SC4OC6/CUR/TMZ (0.85 mM/0.11 mM/0.5 mM) in 10 mM phosphate buffer.

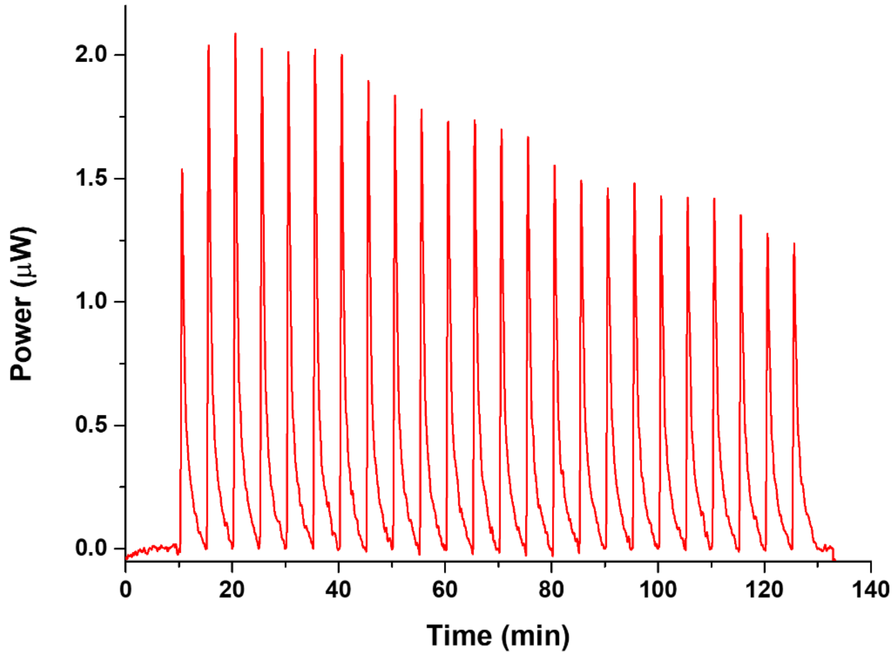

Figure S15. ITC titration curve of TMZ 5 mM into SC4OC6 0.3 mM (above CMC) at 25 °C in 10 mM phosphate buffer.

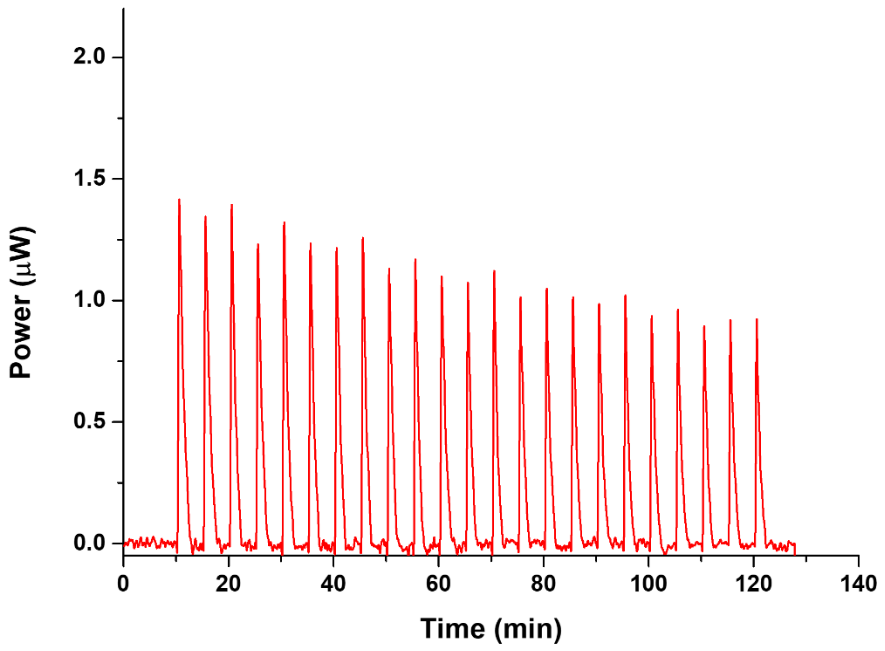

Figure S16. Typical "blank" experiment. ITC titration curve of TMZ 5 mM into phosphate buffer 10 mM at 25 °C.

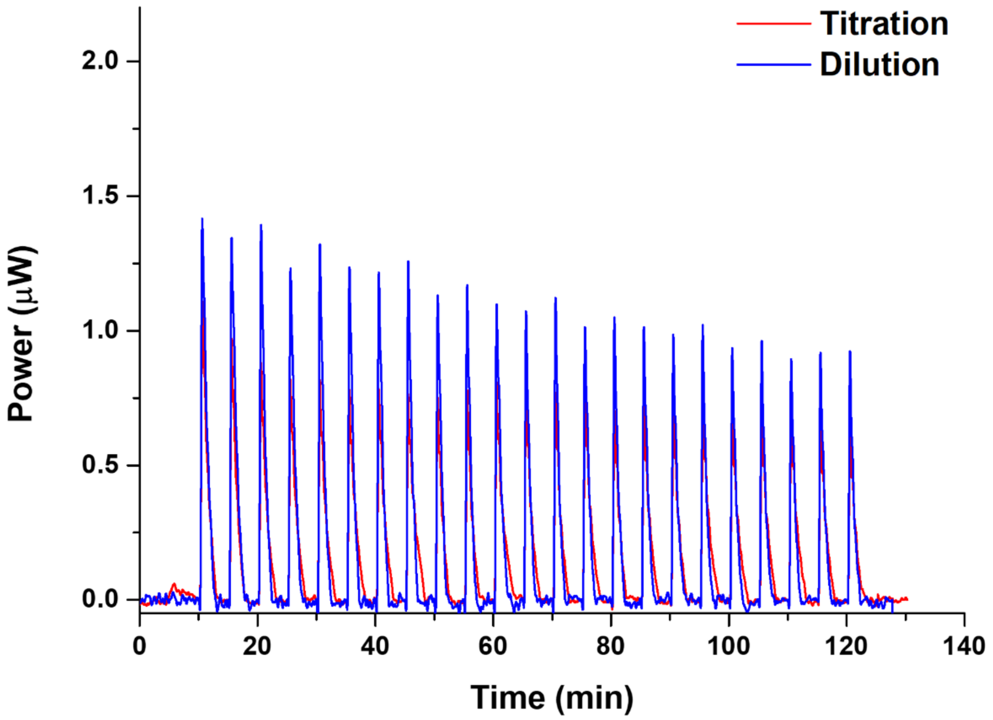

Figure S17. Overlap between TMZ (5 mM) into SC4OC6 (0.02 mM, below CMC) calorimetric titration (red) and the corresponding blank experiment (blue) at 25 °C in 10 mM phosphate buffer.

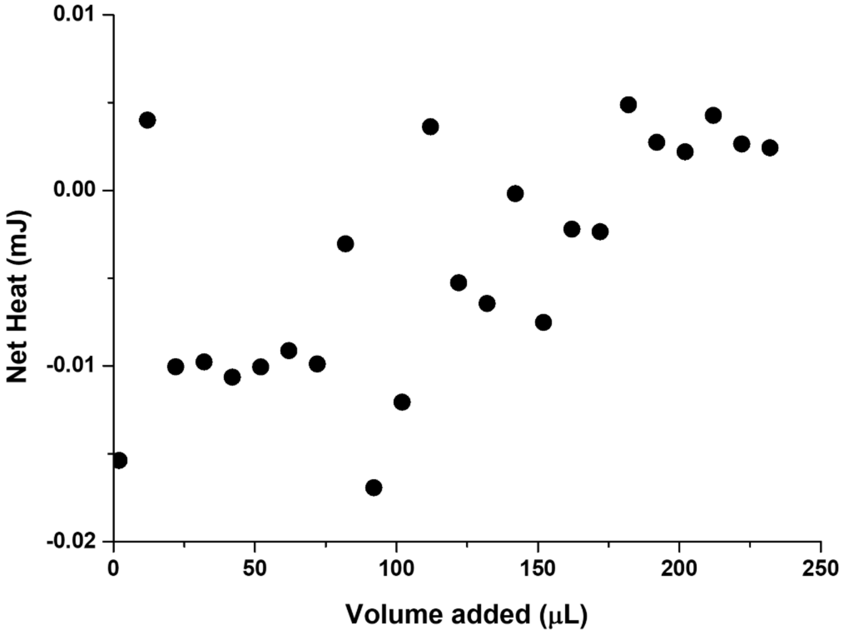

Figure S18. Net heat curve for the TMZ (5 mM) / SC4OC6 (0.02 mM, below CMC) system.

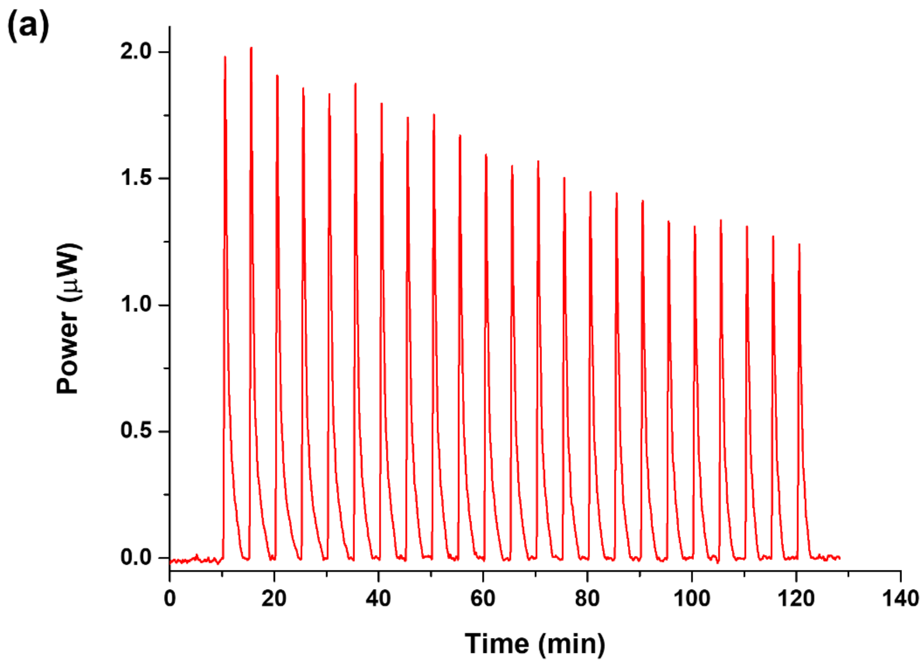

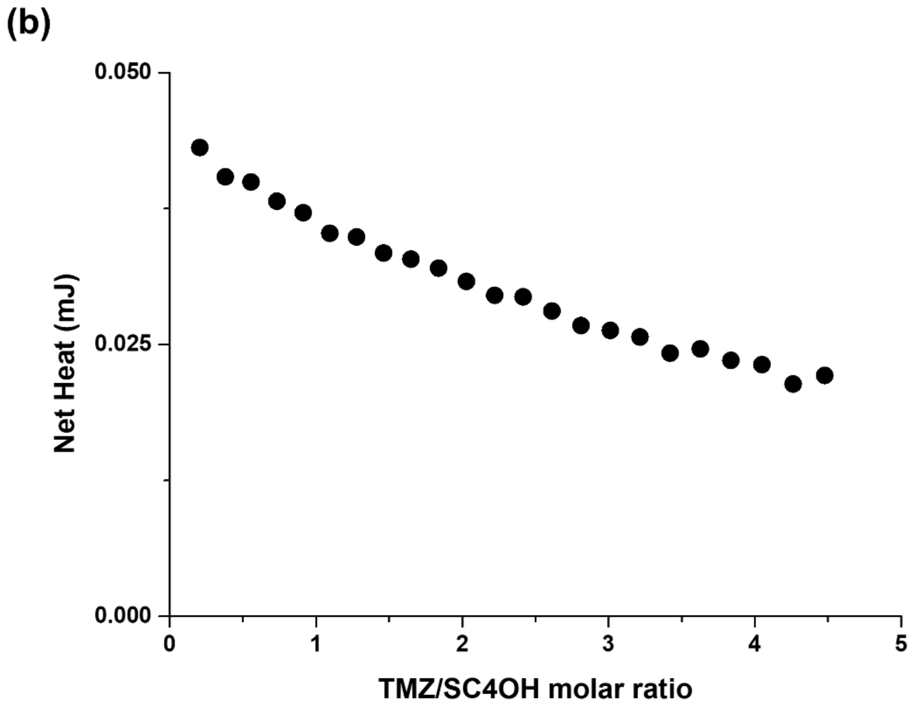

Figure S19. (**a**) ITC titration curve of TMZ 5 mM into SC4OH 0.3 mM at 25 °C in 10 mM phosphate buffer; (**b**) integrated heat data.

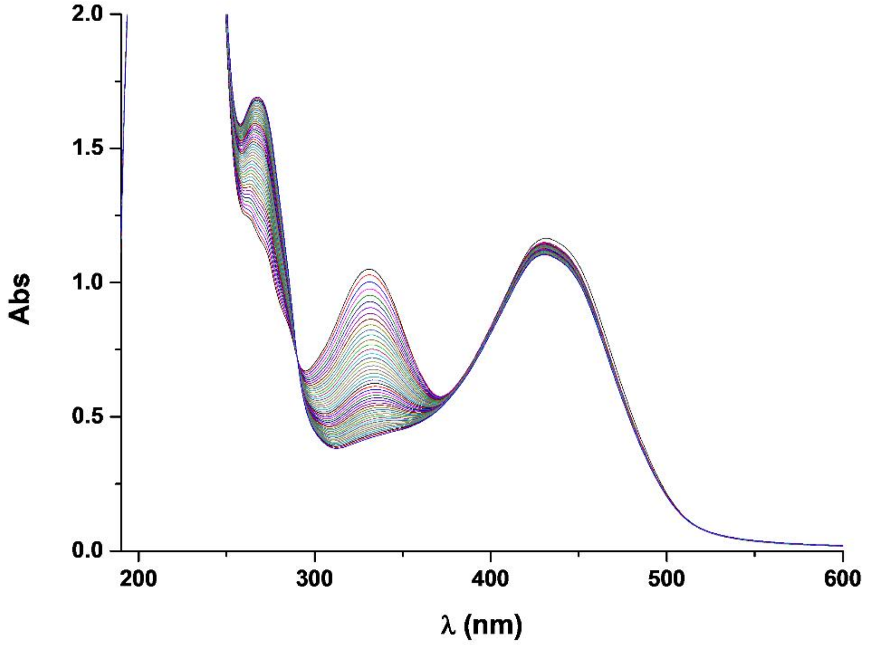

Figure S20. UV-vis spectra of SC4OC6/CUR/TMZ (0.2, 0.025 and 0.1 mM respectively) in 10 mM phosphate buffer recorded every 15 minutes at 37 °C.

Table S3. First order degradation rate constant and half-life values for free TMZ, TMZ-SC4OC6 micelles and TMZ-SC4OC6 micelles loading curcumin in 10 mM phosphate buffer at 37 °C.

	**k (min^{-1})**	**$t_{1/2}$ (h)**
Free TMZ	0.00199 (1)	5.8
TMZ-SC4OC6 micelles	0.00183 (1)	6.3
TMZ-SC4OC6 micelles loading curcumin	0.00139 (1)	8.3
